# Bmal1 integrates circadian function and temperature sensing in the suprachiasmatic nucleus

**DOI:** 10.1073/pnas.2316646121

**Published:** 2024-04-16

**Authors:** Marieke M. B. Hoekstra, Natalie Ness, Aina Badia-Soteras, Marco Brancaccio

**Affiliations:** ^a^Department of Brain Science, Imperial College London, London W12 0NN, United Kingdom; ^b^Department of Brain Sciences, United Kingdom Dementia Research Institute at Imperial College London, London W12 0NN, United Kingdom

**Keywords:** circadian rhythms, temperature sensitivity, suprachiasmatic nucleus, cold-induced pathways

## Abstract

Circadian regulation and temperature dependency are important orchestrators of molecular pathways. How the integration between these two drivers is achieved, is not understood. We monitored circadian- and temperature-dependent effects on transcription dynamics of cold-response protein RNA Binding Motif 3 (Rbm3). Temperature changes in the mammalian master circadian pacemaker, the suprachiasmatic nucleus (SCN), induced Rbm3 transcription and regulated its circadian periodicity, whereas the core clock gene Per2 was unaffected. Rbm3 induction depended on a full Brain And Muscle ARNT-Like Protein 1 (Bmal1) complement: reduced Bmal1 erased Rbm3 responses and weakened SCN circuit resilience to temperature changes. By focusing on circadian and temperature dependency, we highlight weakened transmission between core clock and downstream pathways as a potential route for reduced circadian resilience.

## Significance

Circadian core clock genes transmit 24-h rhythmic information to a plethora of clock-controlled pathways. Rhythmic expression of core clock genes is resilient to thermal changes, a property known as temperature compensation. Here, we show that molecular pathways controlled by core clock genes are not similarly temperature compensated: Increases and decreases in temperature predictably affect their period length. Intriguingly, Bmal1, an essential core clock gene, is required to activate cold-induced transcription of the clock-controlled gene Rbm3, thus suggesting its role as an integrator between circadian function and temperature sensing. The potential rhythmic misalignment between the core clock and its target pathways, induced by temperature, may further reduce circadian proficiency in neurodegenerative diseases.

Temperature affects a vast plethora of biological processes, from cellular metabolism to brain function, which in turn produces heat, further contributing to temperature changes in cells and tissues. Circadian core clock genes show a unique period insensitivity to temperature variations, known as temperature compensation (1). This defining circadian property preserves stable anticipatory timekeeping in the face of a continuously changing temperature input/output balance. At the cellular level, temperature compensation is routed in compensatory molecular network mechanisms within the core transcription feedback loop (TTFL) (2). In contrast, it is not known: 1) whether downstream clock-controlled pathways, also responding to temperature, are temperature compensated; 2) whether the core clockwork plays an active role in sensing and translating temperature changes to downstream clock-controlled pathways, and if so, by which mechanisms.

To investigate this, we chose as a model organotypic slices of the suprachiasmatic nucleus (SCN), the master circadian clock in mammals, because this tissue maintains temperature compensation ex vivo: Temperature changes are ineffective at altering rhythmicity of core TTFL clock genes [reported by PER2::LUC (3)], as opposed to peripheral oscillators (4). This model will therefore allow to compare and contrast how clock-controlled pathways respond to temperature changes, within the context of a self-sustained circadian oscillatory function. Moreover, different ambient temperatures can be easily imposed to these preparations ex vivo, thus ruling out the effects of other behavioral drivers of brain temperature variations, including sleep state (5).

As an elective molecular pathway to investigate how circadian and temperature drivers are integrated, we focused on the cold-induced response of RNA-Binding Motif 3 (Rbm3). RBM3 is an RNA-binding protein previously shown to strengthen the circadian amplitude of core clock genes in immortalized cell lines under temperature-entrained conditions (6). Moreover, induction of RBM3 upon cooling exerts neuro-protectant effects (7), and it is sufficient to revert synaptic and behavioral deficits in mouse models of neurodegeneration (8, 9).

## Results

**Fig. 1.**
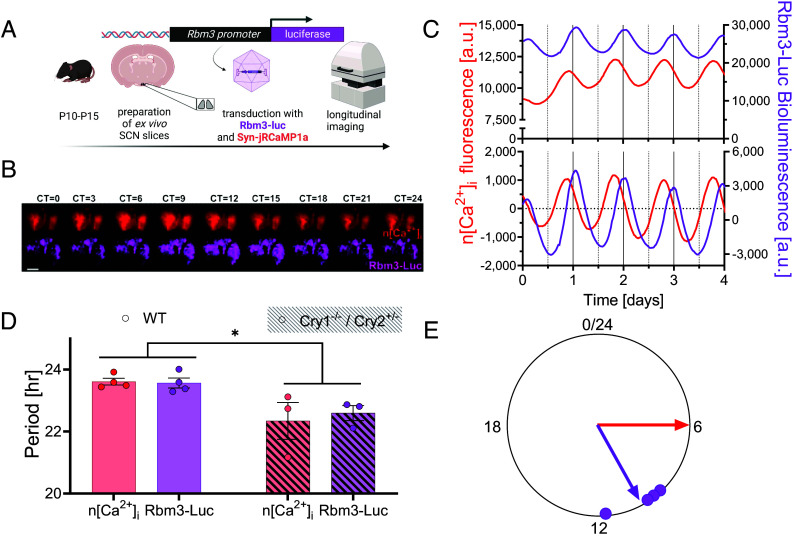
Circadian oscillations of Rbm3-luc expression in SCN organotypic cultures. (*A*) Experimental design. SCN organotypic slice cultures were transduced with Rbm3-Luc and Syn- jRCaMP1a calcium reporters and imaged by multiplexed live imaging. Created with Biorender.com. (*B*) One-day montage with a 3 h time interval of Syn-jRCaMP1a and Rbm3-luc signals. (Scale bar, 200 µm.) See also Movie S1 capturing neuronal Ca^2+^ and Rbm3-Luc circadian expression across the SCN. (*C*) Example of changes in mean intensity in Syn-RCaMP1a and Rbm3-luc of raw data (*Upper* trace) and cubic- detrended (*Lower* trace). (*D*) Period length of Syn-jRCaMP1a and Rbm3-Luc were consistent within genotype (mean ± SEM, n = 4, Syn-jRCaMP1a: 23.4 h ± 0.1 h, Rbm3-Luc: 23.3 h ± 0.2 h; paired two-tailed t test, t(3) = 0.42, *P* = 0.71), but differed across genotypes (two-ways ANOVA, factor genotype, F(1,5) = 8.6, *P* = 0.032). (*E*) Peak phase-time of Rbm3-Luc (circular mean ± SD 10:04 h ± 01:02 h, n = 4) relative to Syn-jRCaMP1a, set at Circadian Time, CT6, based on previous findings (13, 14).

We hypothesized that the integration of circadian and temperature drivers on Rbm3 regulation happens at the level of transcription. To investigate this, we designed a bioluminescence-based reporter of Rbm3 transcriptional activity, by placing a luciferase tag under the control of the Rbm3 minimal promoter (*SI Appendix*, Fig. S1), packaged into adeno-associated viral vectors, AAV-Rbm3-Luc-WPRE (Rbm3-Luc). To characterize circadian dynamics of Rbm3 transcriptional activity, we simultaneously transduced SCN organotypic cultures with AAVs expressing Rbm3-Luc and Syn-jRCaMP1a, the latter being a genetically encoded reporter of neuronal intracellular calcium (n[Ca^2+^]_i_) (10), and imaged them by using multiplexed bioluminescence/fluorescence live microscopy over several days, (Fig. 1*A*). We found robust circadian oscillations of Rbm3-Luc (Fig. 1 *B* and *C* and Movie S1), with a periodicity indistinguishable from codetected n[Ca^2+^]_i_, and peaking about 4 h after (i.e., at circadian time 10, or CT10) (Fig. 1 *D* and *E*). Circadian periodicity of both Rbm3-Luc and n[Ca^2+^]_i_ was shortened by knock-out of Cryptochrome-1 (Cry1) combined with heterozygosity of Cryptochrome-2 (Cry2), as expected for these mutations (11, 12) (Fig. 1*D*). Thus, the transcriptional activity of the Rbm3 promoter is under strong circadian control in SCN slices and predictably altered by deletion of core clock genes (i.e., Cry1^−/−^/Cry2^+/−^).

**Fig. 2.**
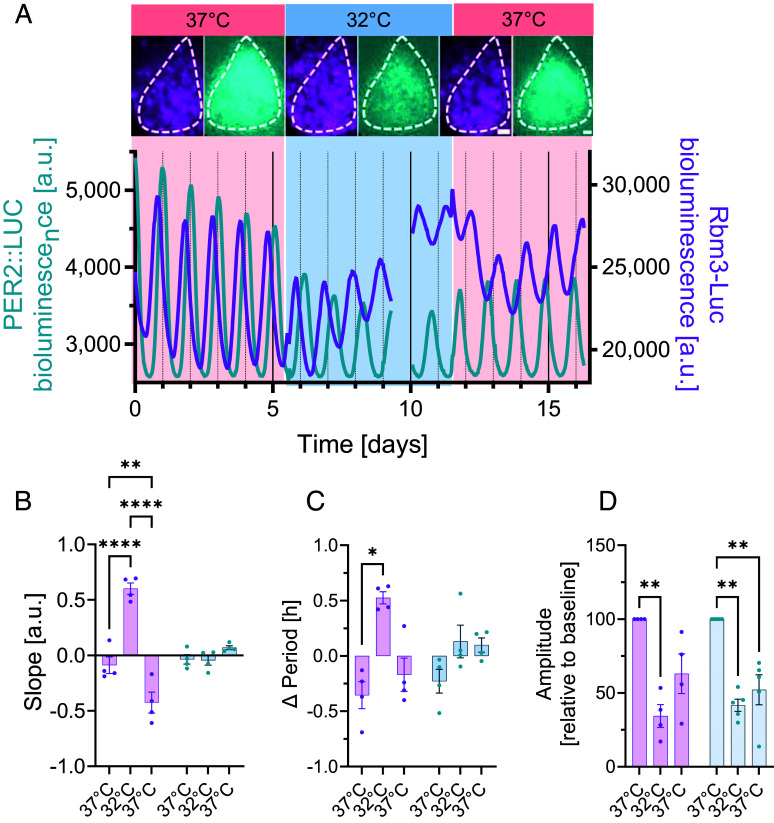
Temperature changes modulate Rbm3-Luc expression and circadian periodicity, but not PER2::LUC. (*A*) Representative SCN slice and bioluminescent traces of PER2::LUC and Rbm3-Luc across different temperature regimes. SCN *Insets* are taken at the through (Rbm3-Luc) and the peak (PER2::LUC) at the end of each temperature phase to demonstrate the temperature- incurred changes in overall intensity. (Scale bar, 50 µm.) (*B*) Significant effect of temperature on overall change in dynamics in Rbm3-Luc, but not PER2::LUC. (*C*) Period lengthening in Rbm3-Luc, but not PER2::LUC at lower temperature, quantified as difference (Δ Period) relative to the overall mean period. (*D*) Circadian amplitude is reduced in both Rbm3-Luc and PER2::LUC reporters. All data plotted are mean ± SEM. (*B* and *C*) Two-ways ANOVA, post hoc Bonferroni’s. *P < 0.05; **P < 0.005; ****P < 0.0001. The gap in recording on day nine is caused by missing values due to technical fault in data acquisition.

To investigate whether Rbm3-driven transcription was also impacted by temperature, we decreased chamber temperature from 37 °C to 32 °C for several days and vice versa and monitored effects on Rbm3-Luc bioluminescence. Temperature reduction drove an immediate and sustained increase in Rbm3-Luc expression, as captured by changes in the slope of Rbm3-Luc expression. Similarly, a return to 37 °C was associated with decreasing levels of Rbm3-Luc. The core clock gene PER2::LUC, sharing the same luciferase tag as Rbm3-Luc remained unaffected by temperature variations, confirming previous findings (4) (Fig. 2 *A* and *B*). Moreover, temperature reductions reduce photon emission of the luciferase/luciferin reporter system (15) and can thus not explain the increase in Rbm3-Luc bioluminescence in cooling conditions and vice versa.

Having shown that Rbm3-Luc expression is determined by both circadian and temperature drivers, we then inspected circadian properties of PER2::LUC and Rbm3-Luc in SCN slices exposed to these different temperatures. While PER2::LUC periodicity remained unaffected by temperature changes, we found that Rbm3-Luc circadian oscillations were lengthened by temperature decrease, and vice versa, shortened by returning to 37 °C (Fig. 2*C*). Lowering temperature decreased the circadian amplitude of both PER2::LUC and Rbm3-Luc (Fig. 2*D*).

Intriguingly, this period mismatch suggests that the periodicity of the core clock (reported by PER2::LUC) and clock-controlled gene expression (reported by Rbm3-Luc) may become misaligned within the SCN in the presence of temperature changes. To reconcile this observation with a shared dependency of both periodicities on elements of the TTFL clockwork, we hypothesized that some core clock genes may also be involved in sensing temperature changes, thereby affecting responses of clock-controlled pathways, including period length. Clock-controlled gene expression will ultimately depend on Bmal1, as the only nonredundant clock gene in mammals (16, 17); moreover, Bmal1 expression changes following temperature variations by both transcriptional and post-translational mechanisms (18, 19). Therefore, we investigated whether Bmal1 is part of a molecular pathway integrating circadian function and temperature sensing of clock-controlled pathways.

**Fig. 3.**
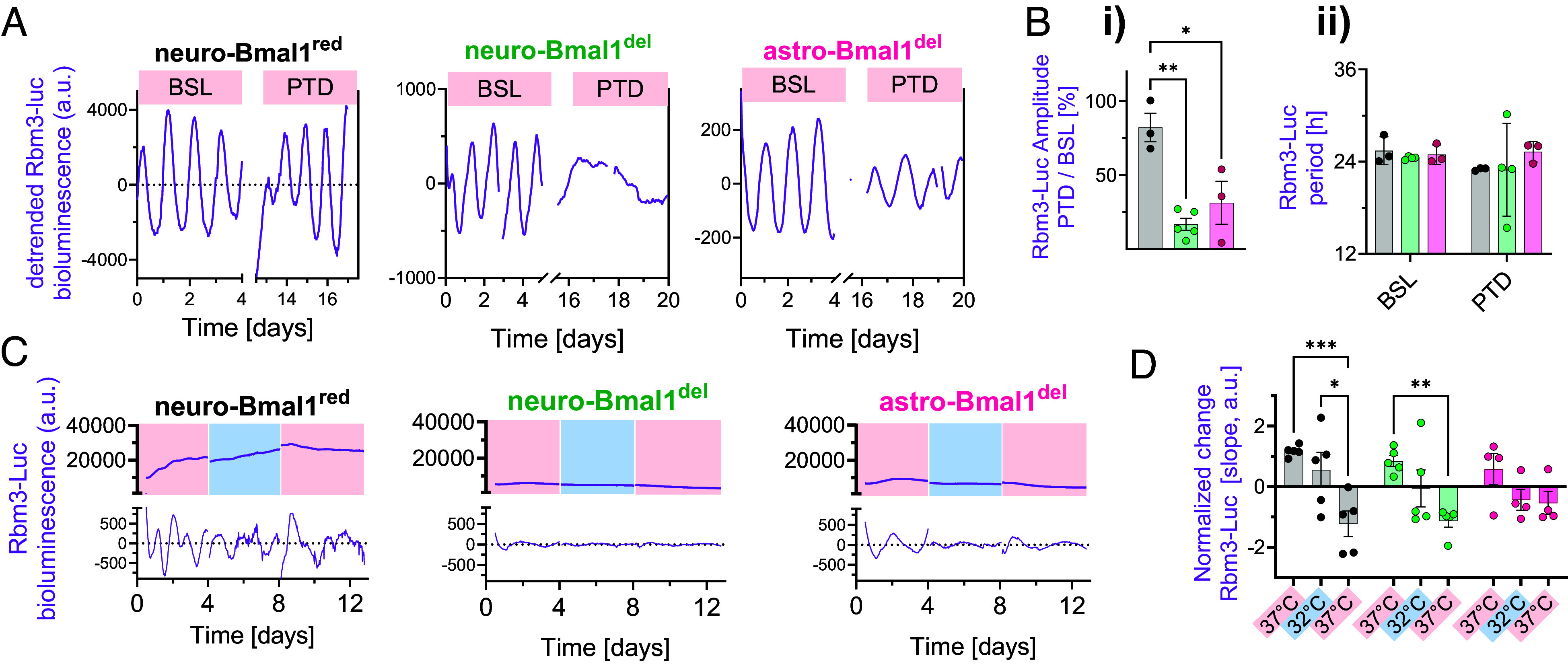
Rbm3-Luc responses to temperature changes are Bmal1-dependent (*A*) Detrended example recordings of baseline (BSL; depicted first 4 d) and eight days post-transduction (PTD). *Left*, black: Bmal1^+/flox^ SCN slices transduced with Syn-Cre::GFP (neuro-Bmal1^red^) (see text); *Middle*, green: Bmal1^flox/flox^ slices transduced with Syn-Cre::GFP, neuro-Bmal1^del^; *Right*: Bmal1^flox/flox^ slices transduced with GFAP-Cre::GFP, astro-Bmal1^del^. (*B*) Relative amplitude (post/pre) of Rbm3-Luc is reduced following treatment in neuro-Bmal1^del^ and astro-Bmal1^del^ SCN slices (one-way ANOVA, *P* = 0.002, Dunnett’s multiple comparisons tests). (*C*) Representative examples of Rbm3- Luc temperature responses in the presence of reduced levels of Bmal1. (*D*) Quantification of Rbm3- Luc transcriptional activity measured by slope change show erased Rbm3-Luc induction in all the models of Bmal1 reduction considered (two-ways ANOVA, post hoc Tukey’s multiple comparison test). Bar graphs represent mean ± SEM, with each data point being an experimental replicate (SCN slice). *P < 0.05; **P < 0.005; ***P < 0.001.

We first evaluated Rbm3-Luc circadian rhythmicity in SCN slices with reduced Bmal1 expression. As both neurons and astrocytes contribute to circadian rhythms in the SCN (13, 20) and express RBM3 (21, 22), we disrupted Bmal1 expression in either of the two cell types separately. We confirmed high colocalization rates of RBM3 in both SCN neurons and astrocytes by using neuronal and astrocytic virally expressed fluorescent tags (*SI Appendix*, Fig. S2 *A* and *B*) and minimal overlap between the Syn-Cre and GFAP-Cre expression (~1%) (*SI Appendix*, Fig. S2*C*). To evaluate whether the circadian rhythmicity of Rbm3-Luc could be specifically ascribed to Bmal1 expressed in neurons or astrocytes, we transduced SCN from Bmal1^flox/flox^ mice (23) with Syn-Cre::GFP or GFAP-Cre::GFP AAVs, to restrict Cre recombinase expression and excise Bmal1 alleles in neurons or astrocytes, respectively. As Bmal1 neuronal heterozygosis is sufficient to sustain circadian rhythms in vivo (24), we also cotreated heterozygote Bmal1^+/flox^ slices with Syn-Cre::GFP AAV, as a way to reduce Bmal1 expression in neurons to a lesser degree (*SI Appendix*, Fig. S3), without affecting overall baseline circadian rhythmicity. Ablation of Bmal1 in both neurons (neuro-Bmal1^red^) and astrocytes (transduced with GFAP-Cre::GFP AAVs, dubbed astro-Bmal1^del^) resulted in a pronounced reduction in Rbm3-Luc amplitude (Fig. 3 *A* and *B*), as opposed to neuro-Bmal1^red^ slices, confirming that i) Bmal1 expression in either neurons or astrocytes is required to mediate strong circadian oscillations of Rbm3-Luc and ii) that robust baseline circadian oscillations of Rbm3-Luc are preserved by reduced Bmal1 neuronal dosage (neuro-Bmal1^red^).

Next, we evaluated whether temperature-evoked Rbm3-Luc responses were altered in neuro-Bmal1^red^, neuro-Bmal1^del^, and astro-Bmal1^del^ SCN slices. After baseline recording at 37 °C, we decreased temperature to 32 °C and after a minimum of 4 d, reverted to 37 °C. In contrast to wild-type (WT) SCNs (Fig. 2), a decrease in temperature did not induce Rbm3-Luc transcriptional activity in any of the experimental groups (Fig. 3 *C* and *D*), thus confirming that full Bmal1 expression in neurons or astrocytes is required for temperature-evoked Rbm3-Luc responses.

**Fig. 4.**
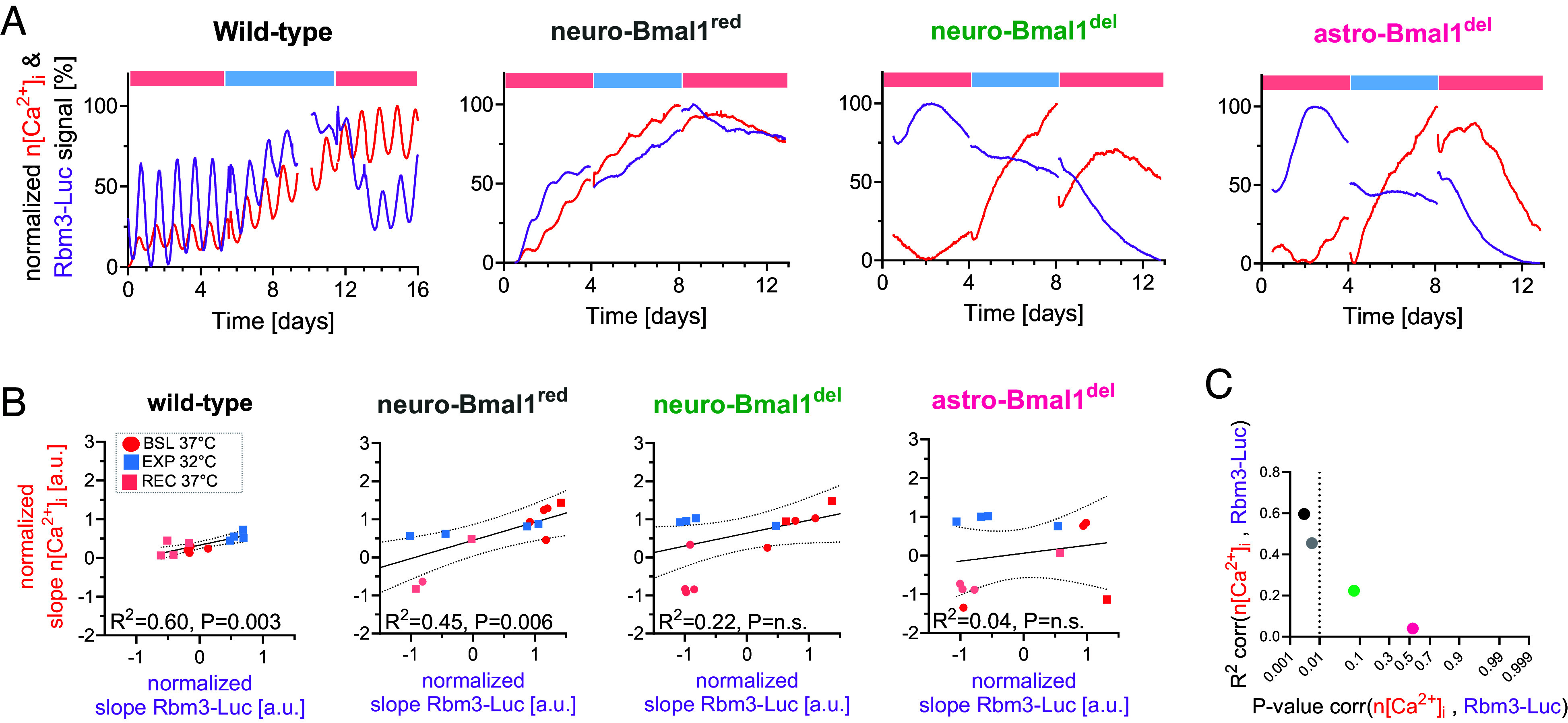
Reduced Bmal1 perturbs the association between Rbm3-Luc and neuronal calcium. (*A*) Representative traces of normalized timeseries of Rbm3-Luc and neuronal calcium coexpressed in SCN slices and evaluated across different temperature regimens. (*B*) Linear regression of the normalized slope of neuronal calcium and Rbm3-Luc across different slices, separated by temperature experimental phase. (*C*) R^2^/p-value plot showing significant correlation between the induction of neuronal calcium and Rbm3-Luc (*P* < 0.01) in both WT and neuro-Bmal1^red^ SCN slices, slightly diminished R^2^ in neuro-Bmal1^red^ and significantly disrupted in neuro-Bmal1^del^ and astro-Bmal1del (low R^2^/p-values).

We next investigated to which extent observations made thus far regarding the integration of circadian function and temperature sensing on clock-controlled Rbm3-luc expression may apply to other clock-controlled processes beyond Rbm3 and gene transcription. Therefore, we assessed how temperature changes affect n[Ca^2+^]_i_ oscillations, as reported by Syn-jRCaMP1a in WT SCN, and compared it with codetected PER2::LUC and Rbm3-Luc, respectively. Neuronal calcium is an essential second messenger, transmitting core clock timekeeping information to a wide palette of clock-controlled biological processes, such as neuronal firing, gene expression, and metabolism (25). We found that a reduction in temperature increased n[Ca^2+^]_i_, similar to what was previously reported (26, 27). The temperature-elicited changes in n[Ca^2+^]_i_ correlated with Rbm3-Luc (Fig. 4 *A* and *B*), but not with PER2::LUC expression (*SI Appendix*, Fig. S4 *A*, *B*, and *D*). Like with Rbm3-Luc, a decrease, or increase, in temperature respectively lengthened, or shortened, periodicity of n[Ca^2+^]_i_ oscillations, but not of codetected PER2::LUC (*SI Appendix*, Fig. S4*C* and Fig. 2*E*), supporting the notion that temperature changes can predictably elicit variations of periodicity in clock-controlled processes in WT conditions.

Because of Bmal1 role in eliciting a temperature-dependent response in Rbm3-Luc expression, we next assessed whether the correlation between n[Ca^2+^]_i_ and Rbm3-Luc was still present in SCN organotypic slices with reduced Bmal1 levels. While the temperature-evoked increase in Rbm3-Luc was abolished by Bmal1 deletion in neurons and astrocytes and by reduced neuronal Bmal1 expression, n[Ca^2+^]_i_ was still induced by temperature reductions (Fig. 4 *A* and *B*). As a consequence, the positive correlation between n[Ca^2+^]_i_ and Rbm3-Luc temperature-dependent induction was weakened (but still present) in neuro-Bmal1^red^ and lost in neuro-Bmal1^del^ and astro-Bmal1^del^ slices (Fig. 4 *B* and *C*). Given that n[Ca^2+^]_i_ acts as a circadian circuit integrator within the SCN (14), and the SCN resilience to changes in temperature is conferred through its network activity (4), we investigated to which degree the Bmal1-dependent change in the relationship between Rbm3-Luc and n[Ca^2+^]_i_ may reflect a weakened organization of the SCN circuitry.

To test this hypothesis, we assessed the circadian spatiotemporal network activity in neuro-Bmal1^red^ SCNs, which retains overall circadian rhythmicity (Fig. 4*A*) and compared it to WT slices. As a readout of the spatiotemporal organization resilience of clock-controlled pathways in response to cooling, we simultaneously monitored Rbm3-Luc and n[Ca^2+^]_i_ rhythmic clusters within the SCN tissue.

To do so, we implemented an unsupervised K-means clustering algorithm, modified from ref. 28, and first validated our analysis on well-established spatiotemporal waves of PER2::LUC expression. Rbm3-Luc rhythms had similar spatiotemporal patterns to PER2::LUC, proceeding from the dorsomedial to the ventrolateral area in the WT SCN, with a comparable intercluster circular variance of phases across the two reporters (*SI Appendix*, Fig. S5). We also confirmed that these spatiotemporal waves of Rbm3-Luc expression were preserved in neuro-Bmal1^red^ SCNs at baseline (37 °C) (*SI Appendix*, Fig. S6).

**Fig. 5.**
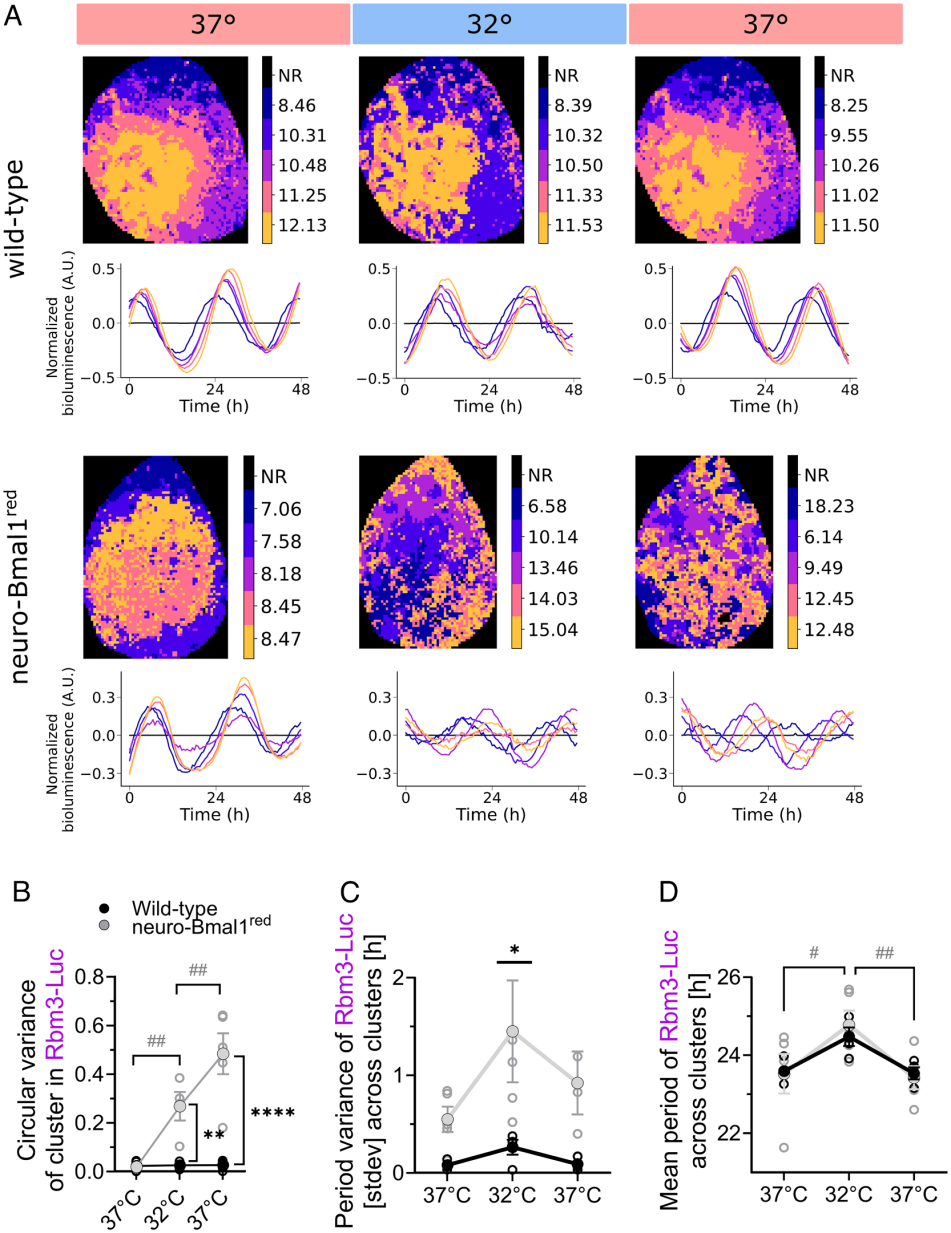
Temperature changes disrupt the spatiotemporal organization of clock-controlled Rbm3-Luc in neuro-Bmal1^red^ SCN slices. (*A*) Representative phase map and traces of clusters reporting circadian Rbm3-Luc spatiotemporal dynamics expressed in wild-type (WT) (*Upper*) and neuro- Bmal1^red^ SCN slices (*Lower*) at 37 °C baseline, 32 °C, and return to 37 °C. Circadian peak phase/cluster is color coded, with NR = nonrhythmic. One SCN nucleus is shown with the dorsal regions at the top and lateral region to the *Left*. Circadian spatiotemporal organization of the Rbm3-Luc clusters is indistinguishable between WT and neuro-Bmal1^red^ SCN slices, but the ability to respond resiliently to temperature changes is specifically lost in the latter. (*B*) Quantification of intercluster phase dispersal measured as circular variance show specific and irreversible increased in phase dispersal of Rbm3-Luc clusters in neuro-Bmal1^red^, but not WT slices (two-ways ANOVA, **P < 0.01, ****P < 0.0001, post hoc Sidak’s multiple comparison test, indicates genotype difference; ##: *P* < 0.01, Dunnett’s multiple comparisons test, indicates difference incurred by temperature within neuro-Bmal1^red^. (*C*) Quantification of period variance (measured by SD) showing increased period variance of the Rbm3-Luc clusters in neuro-Bmal1^red^, but not WT slices (two-ways ANOVA, *P < 0.05, post hoc Sidak’s multiple comparison test). (*D*) Mean period of Rbm3-Luc clusters is affected by temperature both in WT and neuro-Bmal1^red^, suggesting that changes in period observed following temperature variations do not depend on SCN circuit organization (two-way ANOVA, factor “temperature” significant—post hoc Dunnett’s multiple comparison test, ^#^*P* < 0.05, ^##^*P* < 0.01, significant effect of temperature on neuro-Bmal1^red^ SCNs).

Temperature reduction elicited a profound disorganization of the Rbm3-Luc spatiotemporal wave in neuro-Bmal1^red^ (Movie S2), but not in WT SCNs (Movie S3), as reflected by increased intercluster phase (Fig. 5*B*) and period variance (Fig. 5*C*). Increasing temperature back to 37 °C was not effective in restoring spatiotemporal waves of Rbm3-Luc (Fig. 5 *A*–*C*) in neuro-Bmal1^red^. Thus, SCN circuit resilience to temperature changes depends on a full Bmal1 expression. On the other hand, the temperature-elicited changes in mean periodicity of the Rbm3-Luc clusters were present in both WT and neuro-Bmal1^red^ SCNs and indistinguishable (Fig. 5*D*). This confirmed that the temperature-evoked changes of Rbm3-Luc periodicity (Fig. 2) do not depend on reduced Bmal1 expression and consequent circuit-dependent reorganization of Rbm3-Luc clusters and are likely indicative of cellular regulation of periodicity of clock-controlled pathways in response to temperature changes.

The spatiotemporal organization of n[Ca^2+^]_i_ in neuro-Bmal1^red^ fully recapitulated observations made on Rbm3-Luc: it was indistinguishable from WT at baseline (*SI Appendix*, Fig. S6), but cooling specifically disorganized n[Ca^2+^]_i_ clusters in neuro- Bmal1^red^ SCNs, (*SI Appendix*, Fig. S7 *A*–*C* and Movies S2 and S3), thus confirming that SCN resilience to temperature changes is dependent on a full Bmal1 complement. In contrast, mean period elongation was present regardless Bmal1 dosage. Taken together, these results show that while SCN circuit resilience to temperature changes depends on a full Bmal1 complement (as shown by increased phase variance with cooling in neuro-Bmal1^red^ SCNs only), the temperature-elicited changes in mean periodicity of the Rbm3-Luc and n[Ca^2+^]_i_ clusters were present in both WT and neuro-Bmal1^red^ SCNs (Fig. 5*D* and *SI Appendix*, Fig. S7*D*).

## Discussion

Previous studies have focused on the properties of temperature entrainment and compensation of core TTFL function (1, 4). In this study, we extended the investigation beyond the TTFL and examined the effect of interplay between circadian- and temperature- regulation on clock-controlled pathways, by designing a transcriptional reporter for Rbm3 (Rbm3-Luc) regulated by both drivers (Figs. 1 and 2). In contrast to core clock function reported by PER2::LUC, the periodicity of Rbm3-Luc could be predictably modulated by temperature changes (Figs. 2 and 5) which also extended to neuronal calcium (*SI Appendix*, Figs. S4 and S7). Thus, while our data confirm the resilience of clock genes (i.e., PER2::LUC) to temperature variations, it also indicates that periodicity of molecular (i.e., Rbm3 transcription) and metabolic (i.e., n[Ca^2+^]_i_) pathways controlled by the TTFL can be predictably modulated in response to changes in temperature.

We then investigated whether the crosstalk between temperature sensing and circadian regulation of clock-controlled pathways (i.e., temperature-evoked changes of Rbm3-Luc expression levels) may be mediated by TTFL components. BMAL1 protein levels increase following a heat pulse (18), and a subtle (2.5 °C) change in temperature affects Bmal1 transcriptional activity (19), making it a good candidate to mediate such an integration. We first ablated Bmal1 in either neurons or astrocytes (neuro-Bmal1^del^ and astro-Bmal1^del^), as both cell types contribute to timekeeping in the SCN (13, 29). Bmal1 deletion did not only disrupt circadian rhythmicity of Rbm3-Luc (Fig. 3) but also prevented temperature-evoked responses of Rbm3-Luc, irrespective of the cell type in which Bmal1 expression was reduced (Fig. 4).

While these experiments confirmed the role of Bmal1 in temperature sensing and circadian rhythmicity, they did not allow further dissection of their crosstalk, as both functions were simultaneously impaired when Bmal1 was ablated in either neurons or astrocytes. Therefore, we focused on neuro-Bmal1^red^ SCN in which i) neuronal Bmal1 dosage was reduced to a lesser degree (*SI Appendix*, Fig. S3); ii) circadian oscillations of Rbm3-Luc were preserved (Fig. 3), and iii) Rbm3-Luc induction upon cooling was diminished (Fig. 4). Circadian rhythmicity in the SCN emerges from the integration of cell-autonomous properties (e.g., TTFL expression) and circuit-dependent coupling (e.g., synaptic connectivity) (25). To dissect between the two, we imaged Rbm3-Luc and n[Ca^2+^]_i_ clusters within the SCN tissue in wild-type and neuro-Bmal1^red^ and investigated to which degree the effects on circadian rhythmicity observed upon cooling may depend on collective behavior (e.g., reduced relative cluster synchronization contributing to overall period lengthening), indicative of cellular regulation (e.g., period elongation reported in each cluster), or emerging by their reciprocal interactions.

First, we confirmed that reduced neuronal Bmal1 expression granted a spatiotemporal organization of Rbm3-Luc and n[Ca^2+^]_i_ rhythmic clusters, which was indistinguishable from WT controls under baseline temperature, as demonstrated by stable intercluster phase distance (measured by the circular variance) (*SI Appendix*, Fig. S6). The SCN circuit resilience to temperature changes was however specifically lost in neuro-Bmal1^red^ SCNs (but not in WT), as demonstrated by increased phase and period intercluster variance (Fig. 5, *SI Appendix*, Fig. S7, and Movies S2 and S3). Our findings complement the observation that weakened SCN circuit-coupling (by tetrodotoxin) makes the SCN sensitive to phase resetting (4), overall suggesting that the unique resilience of the SCN to temperature changes may depend on the mutual reinforcement of cell-autonomous timekeeping (TTFL-based) and intercellular coupling (mediated by the SCN circuitry).

Notably, however, period lengthening of Rbm3-Luc and n[Ca^2+^]_i_ upon cooling was observed in each of the rhythmic SCN clusters, regardless of Bmal1 expression levels (i.e., in both neuro-Bmal1^red^ and WT SCNs) (Fig. 5*D* and *SI Appendix*, Fig. S7*D*), confirming our observations from whole WT SCNs (Fig. 2*C*). Thus, this effect could not be reconducted to a circuit effect (as intercluster phase and period variance were not affected upon cooling), or a Bmal1 dose effect (as Bmal1 expression was not modified) (Fig. 5 *B* and *C* and *SI Appendix*, Fig. S7 *B* and *C*).

While we investigated n[Ca^2+^]_i_ as a proxy for SCN circuit function, Rbm3 expression may be directly affected by neuronal calcium fluctuations: The Rbm3 promoter harbors calcium-responsive elements (*SI Appendix*, Fig. S1), bound by Calmodulin-dependent protein kinase II/(cyclic AMP/Ca^2+^ response element-binding protein) (CREB), induced by Ca^2+^. Interestingly, the Na^+^/Ca^2+^ exchanger NCX was recently found to mediate cold-induced Ca^2+^ responses via Ca^2+^/calmodulin-dependent protein kinase II in the mammalian SCN, as well as in Drosophila and Arabidopsis (26). Cooling can induce expression of Rbm3 orthologs in several species including plants (30), thus suggesting a potential conservation of this pathway.

The mismatch in periodic responses of both Rbm3-Luc and neuronal calcium (elongation), compared to PER2::LUC (unchanged) upon cooling warrants further investigation. Recently, period dissociation between Bmal1 and neuronal calcium was also found in the SCN during deep hypothermia (27): Low temperatures lengthened the periodicity of neuronal calcium (as in this study). A similar period dissociation, induced by light pulses in vivo, has been observed in Bmal1 and Per1 transcriptional rhythms in the SCN (30). Thus, other studies have also reported that TTFL and downstream clock-controlled pathways may express different periodicities within the same SCN (31); we now suggest that Bmal1 may be a pivotal component in the crosstalk between temperature sensing and regulation of clock-controlled pathways.

Under certain environmental circumstances (i.e., low temperatures), 24-h periodicity of certain clock components (e.g., Per2) may be maintained, while allowing transient disengagement from downstream clock-controlled processes. This disengagement of the clock from downstream processes may mediate adaptive ethological functions, as in arctic animals (32). However, such a disengagement, if protracted, may also become maladaptive, for example, it could exacerbate circadian disruption in neurodegenerative diseases also characterized by perturbed endogenous temperature regulation, such as Alzheimer’s and Parkinson’s diseases (33, 34). Interestingly, the increased death burden of such neurodegenerative diseases has been recently linked to rising global temperatures (35), pointing to the maintenance of endogenous daily thermoregulation as a potential route to disease resilience.

Intriguingly, RBM3 increases upon cooling in physiological conditions [e.g., hibernation (36)], and its overexpression reverts synaptic and behavioral deficits in mouse models of protein misfolding and neurodegeneration (8, 9). RBM3 variations upon smaller temperature variations, such as the ones associated with the sleep wake cycle, could affect clock-gene levels by several mechanisms, including protein translation, and transcript stability by miRNAs binding, and alternative polyadenylation (7, 37, 38). For the latter mechanism, RBM3 contributes to high-amplitude clock-gene transcript levels under temperature-entrained conditions in vitro (6) (*SI Appendix*, Fig. S8). Manipulations of RBM3 levels may offer new insight into the integration of cellular timekeeping and environmental temperature cues, in both physiological and pathological conditions.

## Materials and Methods

### Animal Experiments.

Experiments on SCN samples were carried out in accordance to regulations of the UK Home Office under the Animals (Scientific Procedures) Act 1986, with Local Ethical Review by the Imperial College London Animal Welfare and Ethical Review Body Standing Committee (AWERB). Mouse strains used were PER2::LUC (B6.129S6-Per2tm1Jt/J), RRID: IMSR_JAX:006852, and Cry1^−/−^/Cry2^−/−^ mice, both gifts from Michael Hastings, MRC-LMB, Cambridge; B6.129S4(Cg)-Arntltm1Weit/J (Bmal1-flox), RRID: IMSR_JAX:007668, gift from Simone Di Giovanni, Imperial College London.

### Ex Vivo SCN Preparation.

Male and female mouse pups (P10-P15, C57Bl6/J background) were killed to extract their SCN, as in ref. 14. Briefly, after isolating the brain and placing it in cold dissection medium [GBSS (Sigma G9779) supplemented with: 5 mg/mL glucose (Sigma 158968), 100 nM MK801 (Sigma M107), 3 mM MgCl2 (Invitrogen AM9530G), 50 µM AP-5 (Tocris 0106); filtered with steriflip 0.22 µm pore size], the lateral and dorsal segments of the brain were removed, exposing the medial-ventral part of the brain slices cut at 300 µm by using a tissue chopper (McIlwain Tissue Chopper). The slice containing SCN tissue was selected, and non-SCN tissue was cut off after which the remaining SCN tissue was placed on a membrane [Merck PICM0RG50] on initial plating medium for approximately 3 h [air medium working solution, supplemented with 100 nM MK801, 3 mM MgCl2 and 50 µM AP-5; air medium stock solution recipe: 500 mL ddH2O with 4.15 gr DMEM (Sigma D5030), 0.175 gr NaHCO3, 2.25 gr glucose (Sigma 158968), 5 mL penicillin/streptomycin (Sigma P4333), 5 mL HEPES 1M (Sigma H0887), for working solution, supplemented with 5% fetal bovine serum (Gibco 10270106), 1% B27 (Gibco 17504044) and 0.5% Glutamax (Invitrogen 35050- 038)]. Three hours later, the membrane was transferred to air medium working solution and kept sealed in temperature-controlled conditions (37 °C). Slices were placed every week in a new six-well with fresh medium.

### AAV Transduction.

At least 1 wk after slice preparations, thereby allowing the cultures to settle, SCN slices were transduced by dropping 1 µL to 1.2 µL of concentrated AAV suspensions onto the slice (AAVs titer was at ≥ 1 × 10^13^ GC/mol). At least 5 d were left in between this and successive AAV transductions. For transduction of Bmal1^flox/flox^ and Bmal1^+/flox^ slices in Fig. 3 without temperature change, the following imaging and transduction protocol was used: Baseline circadian rhythmicity of Rbm3-Luc expression was monitored for 4 d (BSL) and compared longitudinally in each SCN slice with the one recorded 8 d post-AAV transduction (PTD) after a medium refreshment. For transduction of Bmal1^flox/flox^ and Bmal1^+/flox^ with temperature change, slices were transduced with the CRE-AAV at least 5 d before the start of the baseline (37 °C). AAVs used were rAAV8/GFAP-Cre::mCherry, rAAV8/Syn-Cre::GFP (Vectorcore, UNC); pAAV1 [Exp]-{RBM3promoter}>Luciferase:WPRE, designed in house (Vectorbuilder id.: VB200519-1118txj); pAAV.Syn.NES.jRCaMP1a.WPRE.SV40 (Addgene id.: 100848).

### Bioluminescence/Fluorescence Multiplexed Time-lapse Imaging.

A LV200 microscope (Evident/Olympus) mounted on a motorized stage was used with a 40× long- distance range objective to perform live imaging experiments. Imaging settings (light intensity, exposure time and EM gain) were kept constant throughout each experiment. Slices were placed in glass-like bottom six-well plates (CellVis, P06-1.5H-N) with 1.2 mL air medium supplemented with 100 nM luciferin (AAT Bioquest) and sealed with a film (Sigma Aldrich, Z369667) to prevent medium evaporation. Temperature was controlled with a TOKAII HIT, and the temperature of the top-plate was kept at +1 °C relative to the other components to prevent condensation.

### Data Analysis of SCN Live Imaging Recordings.

#### Image processing time-lapse images.

Raw data files were converted into .tiff files, and motion correction was applied to slices that moved slightly across the *x*–*y* axis during the recording (FIJI plug-in, Descriptor-based series registration 2d/3d+t; ref. 39.

#### Quantification of temperature-induced changes of reporter expression.

To control for variation in signal intensity, the data were normalized where the largest value was 100%, and the smallest value 0%. Then, a linear trend was fitted through the data. The slope of this linear line is reflective of overall changes in the temperature-dependent dynamics of the reporter (Graphpad Prism v9).

#### Extracting circadian parameters.

After extracting the region-of-interest, the SCN, in FIJI, the mean intensity trace is analyzed in Biodare2 (40) (https://biodare2.ed.ac.uk/). The signal was cubic detrended after which a nonlinear least squares fast Fourier’s transformation is applied to extract circadian parameters of the dominant sinewave in the data. To assess phase (peak time), the “Circ. Phase To Zero” variable was extracted.

### Statistical Analysis.

All statistical analyses were conducted in Prism version 9, with “n” referring to the number of independent slices analyzed. To compare across different experimental groups, significance was tested. First, the distribution of the data was assessed: If the data were normally distributed, a parametric test was applied (e.g., *t* test, ANOVA), if not, a nonparametric test was used (e.g., Mann–Whitney *U* test). If experimental data consisted of more than two groups, post hoc tests were applied. The threshold of significance was set at α = 0.05, and specific statistical tests used are mentioned in the figure legends.

### K-Means Clustering of Timeseries.

A moving average was applied to the image series (window: 24.5 h), using the linear stack detrend tool by Jay Unruh at the Stowers Institute for Medical Research in Kansas City, MO in FIJI. Images were divided into rectangular regions of interest of 3 × 3 pixels covering the SCN. Timeseries were extracted from each region of interest. For the K-Means clustering, we used a modified approach from ref. 28. Using custom Python script, the timeseries of each region of interest were normalized and a K-Means clustering algorithm was implemented using the K-Means algorithm from scikit-learn 1.2.2 (41) with k = 5 and the classical EM-style Lloyd algorithm. Circadian parameters of the mean timeseries of each cluster were then calculated as described above, and parameter “Abs. Phase To Zero” was used to determine peak time per cluster, to control for any period effects. Cluster maps and timeseries graphs were created using the matplotlib package (42). All SCN phase maps show a single nucleus with the dorsal SCN at the top, medial to the right, ventral to the bottom, and lateral to the left. Vectors of spatiotemporal progression of the clusters were calculated by linear regression of the center of mass of the clusters, using the center of mass function from the scipy multidimensional image processing package scipy.ndimage (43), followed by fitting a linear regression using scikit-learn 1.2.2v (41).

The circular variance of cluster phases or vector angles was calculated as follows:

$$\mathrm{Circvar}=1-\frac{\sqrt{(\sum \text{sin}\left(x\right){)}^{2}+(\sum \cos(x){)}^{2}}}{n},$$

where x is the phases or vector angles in radians and n is the number of phases or vector angles.

### Immunohistofluorescence on Fixed SCN Slices.

#### Staining.

SCN slices in *SI Appendix*, Fig. S2 were transduced 1 wk after preparation with a 1:1 mix of 1.2 µL AAV8-Syn-Cre::GFP and AAV8-GFAP-Cre::mCherry, to label Syn^+^ and GFAP^+^ cells, respectively. At least a week after transduction, slices were fixed with 4% PFA over 2 h at room temperature at day 10 post-transduction and incubated for 1 h with 10% donkey serum (Abcam, ab7475) in day 1 buffer (1× PBS, 1% bovine serum albumin and 0.3% triton-X) at room temperature. Slices were incubated overnight at 4 °C with primary RBM3 antibody 1:1,000 (Abcam, ab134946) and washed twice for 5min with day 2 buffer (1/3 dilution of day1 buffer in 1× PBS), followed by a 1 h incubation at room temperature with a 1:1,000 secondary antibody donkey anti-rabbit 647 (Abcam, ab150075) in day 2 buffer. Slices were washed twice for 5 min in day 2 buffer, followed by 3 × 20 min washes in 1× PBS, and mounted on a glass slide after which they were covered with mounting medium (Prolong Glass with NucBlue, P36985) and a coverslip. SCN slices in *SI Appendix*, Fig. S4 were transduced with AAV8-Syn-Cre::GFP, followed by weekly medium refreshment and fixed after no less than 3 wk after with 4% PFA. Staining procedure as above, while using a Bmal1 antibody (Novus Biological, NB100-2288) instead of Rbm3. Slices were imaged at a confocal microscope, SP8 Leica with 20× objective, using the same settings across conditions per antibody staining.

#### Analysis RBM3 staining.

GFP^+^, mCherry^+^ and RBM3^+^ cells were identified by applying thresholds, followed by a particle analysis to identify soma that were positive for the above markers. Overlap of GFP^+^/mCherry^+^, and GFP^+^/RBM3^+^ and mCherry^+^/RBM3^+^ was manually determined.

#### BMAL1 staining.

BMAL1^+^ cells were determined by applying a threshold. These data were used to identify BMAL1+ soma, of which subsequently the total area was identified and plotted relative to the whole area on which the thresholding was applied. Images in *SI Appendix*, Fig. S4 represent raw data.

## Supplementary Material

Appendix 01 (PDF)

Movie S1. **Circadian oscillations of Syn-jRCaMP1a and Rbm3-Luc monitored in SCN slices.** Left: neuronal calcium, right: Rbm3-Luc. Sample is imaged every 30 minutes for five consecutive days at 37°C.

Movie S2. **Related to Figure 5 and S5. Reduced Bmal1 expression irreversibly impairs spatiotemporal organization of Rbm3-luc and SCN circuit activity with temperature changes.** Neuro-Bmal1^red^ shows normal circadian spatiotemporal organization of neuronal calcium and Rbm3-Luc during four days of baseline recording at 37°C, which this is impaired when temperature is decreased to 32°C. Reverting temperature back to 37°C does not restore the spatiotemporal organization of neuronal calcium and Rbm3-Luc signals.

Movie S3. **Related to Figure 5 and S5. Change in temperature does not affect spatiotemporal organization in wild-type SCN slices.** In contrast to the neuro-Bmal1^red^ sample, spatiotemporal organization of the SCN circuit is preserved with changes of temperature in WT SCN slices

## Data Availability

All study data are included in the article and/or supporting information.
