# Supplementary material for: Bmal1 integrates circadian function and temperature sensing in the suprachiasmatic nucleus
Source: Proc Natl Acad Sci U S A. 2024 Apr 16;121(17):e2316646121. doi: 10.1073/pnas.2316646121 (PMC11047078; doi:10.1073/pnas.2316646121)
Supplement: Supplementary file 1 — Appendix 01 (PDF) [file pnas.2316646121.sapp.pdf]

# Supporting Information for

# Bmal1 integrates circadian function and temperature sensing in the suprachiasmatic nucleus.

Marieke MB Hoekstra[1,2], Natalie Ness[1,2], Aina Badia-Soteras[1,2], Marco Brancaccio[1,2*]

[1]Department of Brain Science, Imperial College London, London W12 0NN, United Kingdom.

[2]United Kingdom Dementia Research Institute at Imperial College London, Department of Brain Sciences, London W12 0NN, United Kingdom.

*Corresponding author
**Email:** m.brancaccio@imperial.ac.uk

*This PDF file includes:*

Figures S1 to S8
Legends for Movies S1 to S3
Supplementary References

*Other supporting materials for this manuscript include the following:*

Movies S1 to S3

TGCTCCAGGCTCCCCAAGTGCTAGGATTTCTGGTCTAAGTTATCATACCCAACTAAAGTGCCAGATCTTAATGG
GAACAGTATCAGTGTGCACATACCTTAAACCCTCACGTAATATGACGTTATTCTCAACTCTACATTAAATGCAA
TTTCAAAAATCATCCAATTTTTGTTTGCTTATTTGAATACGGGGTCTCACCATAGGCTAGCTAAAGTTAGCCTGG
AACTTGTTAAAGCTCCAATTCACAACCCTGCCTCAGTTTTCTGATAGATGGTGATGAGGCATCTGGTTCAATGG
CACAGTAAAAGCAGACACAGCTCTCTCTCTTTGTTGGGATATAAGCCAAGGGACTTGCACATACTAGGAAGGC
ACTCTGCCACTGAGCTGAACCCTATAGAAGGACTGAGGACCTTAAACTAAAAGCAAAAGTATACAGCTAACGT
AATAAGCTGCAGAAGAGTATCTTTGGGACCTGGGCAGGGAGGATTTCTTTAAAAGTACAGACACGCTGGTTG
TGGTGATGATCCCAGCACTGAAGAGGCTGAGGCAAAAGTTTTATGAGTTCCAGGGTGACCTGAGCATACAGT
GAGGGACAGACCACAGGGTGAGAAATATTAGAACAACAAAAAGTATTCAACTAAAGATAACTACTTACAAAT
TTAATAAAAATCCATGAAAATCACTGAGAAATGTGGGAATAAGCTTACCAGGAATTACAGATGAATGGCATCC
AAAGGGTAGCAAATCGATTTGTGGTGGGGTTGGTGGAGGAGTGGGAGGCAGGCCTTTACAATGTTGAACAA
ATTTATTCTAGGAGTCAACAATGTAGAGTTCCTGGAGGCAAACAAGTTATTAGGCACTGAAGGCTGGGAAAG
ATCACATCGTCAGGCAGTGGACTTAGACACTACTTGCTATGGATTTCATTATGTACTGCAAAAGTTCATCCTTTC
GATCAAGACCCAGTATCCCGAATAGAGAGTAAAATTCGTTTAGTGGATTGAGATTTGCATCCTTTGAAGCTAG
GCTACCTGGCTTGCTGAACGCATTATCAGGCGTAAAGAGACTG**TCAGATAAGGA**TGCAAACTTAAGTCAGAG
GATGACACCAAGGGAAGGAGTCCGCTAACACACAGCTATATGTTAGGAGGAGGGGCTAAGCATTCGGAAAT
GTAAGGTTTGACAGGAAGAACTCTGAAGGCCTCGGGGCCTTAAGGGATATGCAGTTAAAGGTCTAAAAAGAA
AAGATTGGTTTCTGAGGACGGCAGTTCGCTGGCACCGACCTACTGTCAGTTAGGACTTCCTATGGAAAGGGA
GAGATGACGTCAAAAACACTGAAAATGACGTCAGGGAGGAAAAGGCTCTATGGATCCAACCGTTTGAGATGG
AAAGGGGGCCAGTCTCTCGGGCAGAAAGGAGATGCTTATAGGAGAACGCACTGAACTCATAAAGCTCCTC**TC**
**GATTCCCGGAAG**CGGCGAGGCAAATTGCTGCCTCCGCCTTCCTCGTCCTCTTCCCAACCCCCCCCCCCCGCTGG
CCTCAACCAATGAGCACGCACGCCAGGGCGCGCTGGGGGACGTTGTGTGACGTCTCGGCTACGCACGCTTTA
TCAGGCGTC**TTCCCGCGCCG**C

**Figure S1. Rbm3-luc promoter sequence.** Sequence of Rbm3 promoter in AAV Rbm3-Luc. Gene modulatory elements are highlighted as follows. cAMP responsive element (CRE): TGACGTCA. Binding site for transcription factors: E2F3, Stat4, Gata1. No matches were found with E-boxes.

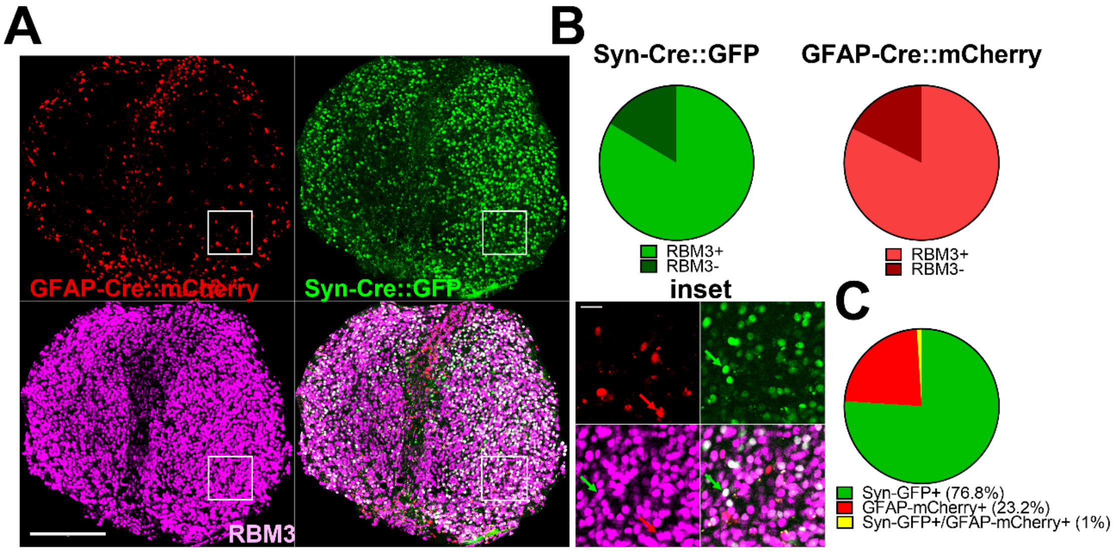


**Figure S2. RBM3 protein colocalizes with GFAP-Cre::mCherry and Syn-Cre::GFP. (A)** example of SCN slices co-transduced with AAV-GFAP-Cre::mCherry and AAV-Syn-Cre::GFP, with RBM3 detected with immunohistofluorescence (LUT, magenta). Red arrow indicates an example of GFAP-Cre::mCherry$^+$ and RBM3$^+$ cells, green arrow indicates Syn-Cre::GFP$^+$ and RBM3$^+$ cells. **(B)** The majority of Syn-Cre::GFP$^+$ cells express RBM3 (mean 83.5%, SD 11.5%), as do GFAP-Cre::mCherry$^+$ cells (mean 82.3%, SD 6.9%).(C) Pie charts showing minimal overlap between Syn-Cre::GFP and GFAP-Cre::mCherry expression (mean±SD, GFP$^+$ cells: 76.8±18.7%, mCherry$^+$ cells: 23.2±18.7%, GFP$^+$/mCherry$^+$ cells: 1.0±0.8%). N=4 SCN slices. Scalebar= 250µm, scalebar inset= 50 µm

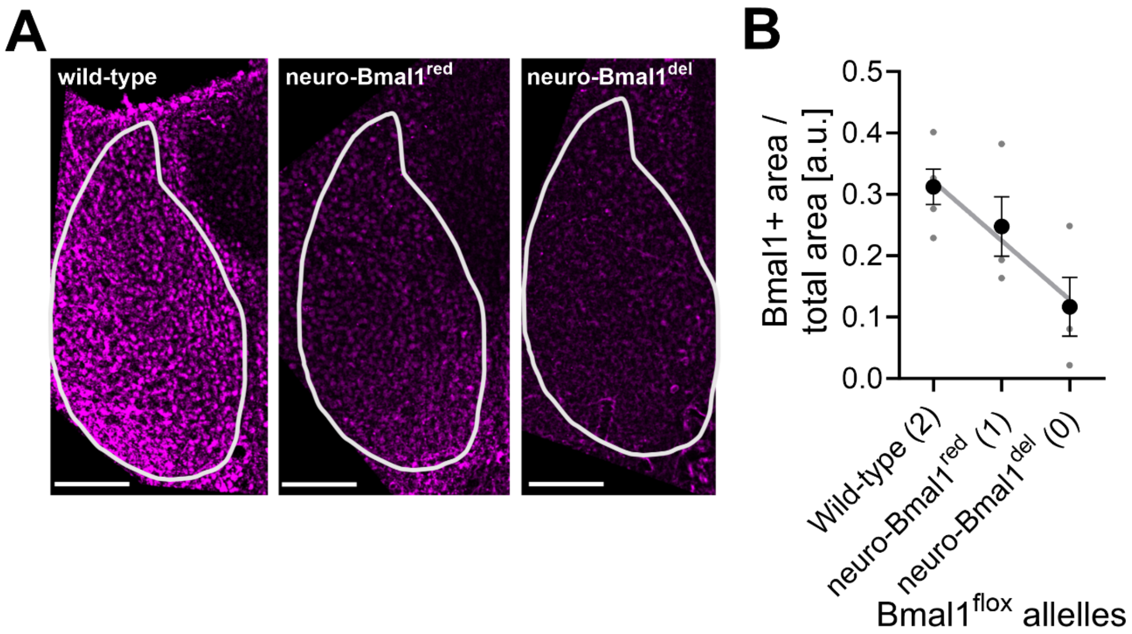


**Figure S3. Decreased Bmal1 expression in neuro-Bmal1red and neuro-Bmal1red SCN. (A)** Representative images of SCN slices transduced with Syn-Cre expressing AAVs showing BMAL1 expression detected by antisera in wild-type, neuro-Bmal1red and neuro-Bmal1del animals. **(B)** quantification of Bmal1+ area relative to the total area analyzed, showing a significant effect of Bmal1flox genotype on Bmal1 expression. Regression line shows significant dose-dependency of reduced Bmal1 expression with reduced number of Bmal1 genomic alleles ($R^2$=0.52, P=0.005). $n_{scn}$ =4, 5 for each genotype. Scalebar=100µm.

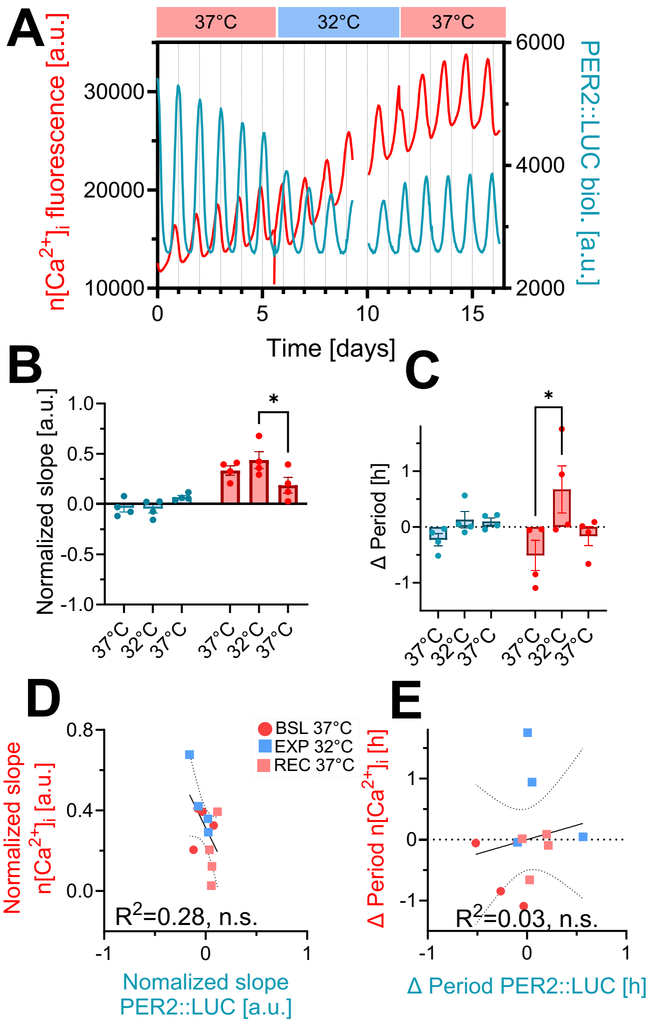


**Figure S4. Differential temporal evoked responses in neuronal calcium and PER2::LUC. (A)** Representative traces of Syn-jRCaMP1a reporting neuronal calcium and co-detected with PER2::LUC reporting core clock gene expression. **(B)** slope of PER2::LUC (cyan) and neuronal calcium (red) (2-way ANOVA, post-hoc Bonferroni's test). **(C)** Change in period evoked by temperature is significant in neuronal calcium, but not PER2::LUC. Temperature-evoked changes in slope **(D)** and period **(E)** of neuronal calcium do not correlate with changes in slope and period of PER2::LUC. Bar graphs are mean±SEM, *= P<0.05

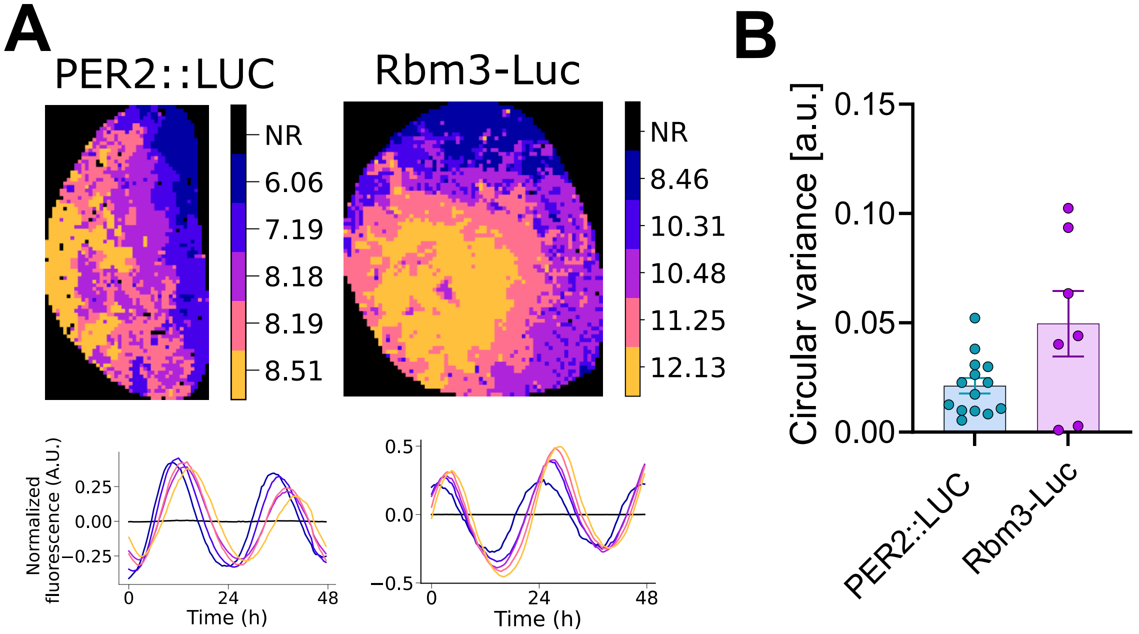


**Figure S5. Phase maps of PER2::LUC and Rbm3-Luc spatiotemporal organization in SCN slices. (A)** Representative phase maps and traces reporting cluster organization in SCN slices respectively expressing PER2::LUC, or Rbm3-Luc reporters. One SCN nucleus is shown with the dorsal regions at the top and lateral region to the left. Phase maps show similar medial-lateral to ventral-lateral spatiotemporal progression across the two reporters. Circadian peak phase/cluster is color-coded, with NR= non-rhythmic. **(B)** Mean inter-cluster phase dispersal within PER2::LUC and Rbm3-Luc clusters as reported by mean circular phase variance is indistinguishable across the two reporters. Bar graphs: mean±SEM, unpaired Welch's t-test, t(6.7)=1.8, P=0.11

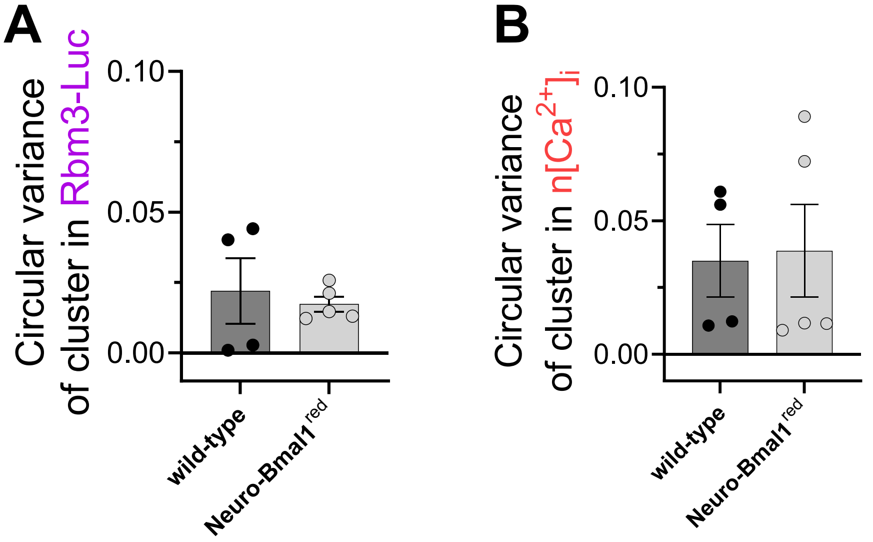


**Figure S6. SCN circuit organization is not affected by Bmal1 reduction at baseline temperature.** Circular variance of Rbm3-Luc **(A)** and neuronal calcium **(B)** clusters under baseline conditions in wild-type compared to neuro-Bmal1$^{red}$ slices compared to WT. The spread of the clusters does not significantly differ between the two groups (Mann-Whitney U test).

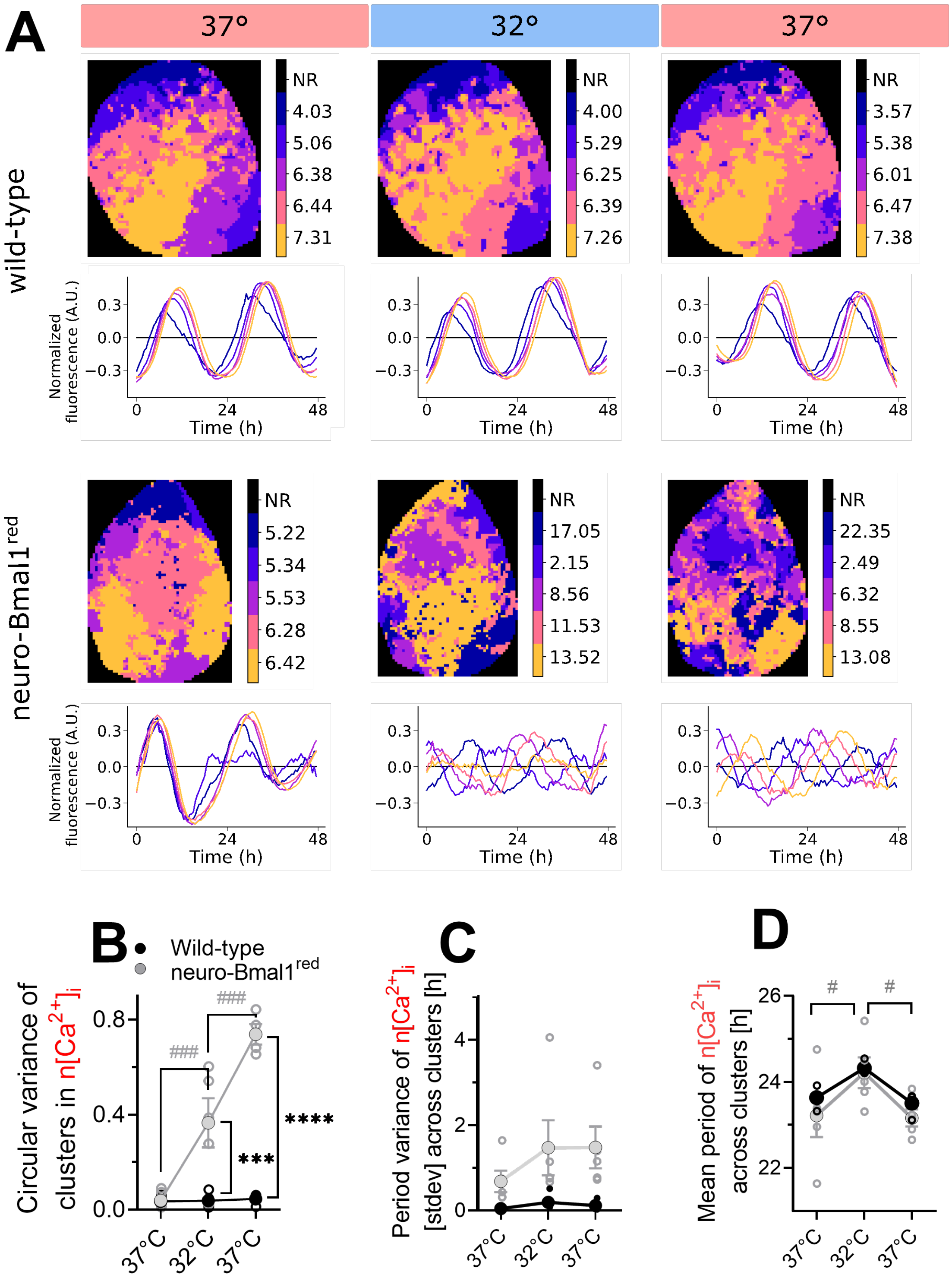


**Figure S7. Temperature changes disrupt the spatiotemporal organization of intracellular calcium in neuro-Bmal1red SCN slices. (A)** Representative phase map and traces of clusters reporting circadian Syn-jRCaMP1a (reporting intracellular neuronal calcium) spatiotemporal dynamics expressed in wild-type (upper) and neuro-Bmal1red SCN slices (lower) at 37°C baseline, 32°C and return to 37°C. Circadian peak phase/cluster is color-coded, with NR= non-rhythmic.

One SCN nucleus is shown with the dorsal regions at the top and lateral region to the left. Circadian spatiotemporal organization of the Syn-jRCaMP1a clusters is indistinguishable between WT and neuro-Bmal1$^{red}$, but their ability to respond resiliently to temperature changes is specifically lost in neuro-Bmal1$^{red}$. **(B)** Quantification of inter-cluster phase dispersal measured as circular variance show specific and irreversible increased in phase dispersal of Syn-jRCaMP1a clusters in neuro-Bmal1$^{red}$, but not wild- type slices (2-ways ANOVA, ***= P<0.005, ****= P<0.0001, post-hoc Sidak's multiple comparison test, indicates genotype difference; ###: P<0.005, Dunnett's multiple comparisons test indicates post-hoc difference incurred by temperature within neuro-Bmal1$^{red}$. (C) Quantification of period variance (measured by SD) show increased period variance of the Syn-jRCaMP1a clusters in neuro-Bmal1$^{red}$, but not WT slices (2-ways ANOVA, factor Genotype, P=0.002) which does not reach significance post-hoc. (D) Mean period of Syn-jRCaMP1a clusters is affected by temperature both in WT and neuro-Bmal1$^{red}$, suggesting that changes in period observed following temperature variations do not depend on SCN circuit organization (2-ways ANOVA, factor "temperature" significant, post-hoc Sidak's multiple comparison test only reaches significance in neuro-Bmal1$^{red}$, #= P<0.05)

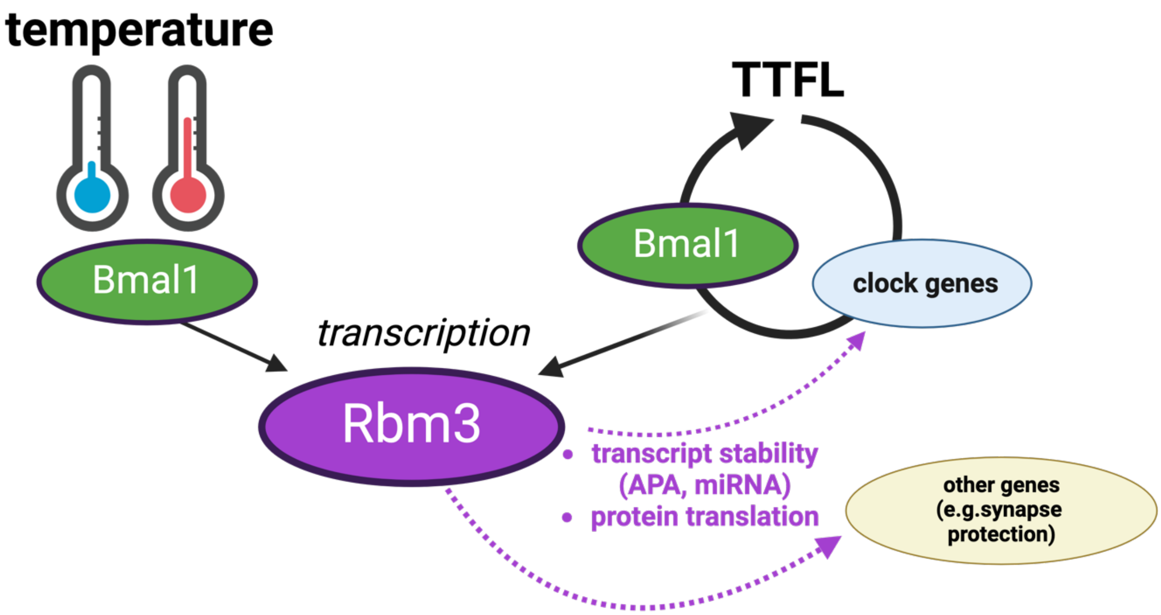


**Figure S8. Summary of putative mechanisms of Rbm3-dependent regulation of circadian function**
Cooling increases RBM3 protein levels, which have been shown to influence gene expression levels via various mechanisms including: i) transcript stability via alternative location of poly-adenylation tail (APA) (1) and increased microRNA levels (2), and protein translation (3, 4). Created with Biorender.com

**Supplementary Movies**

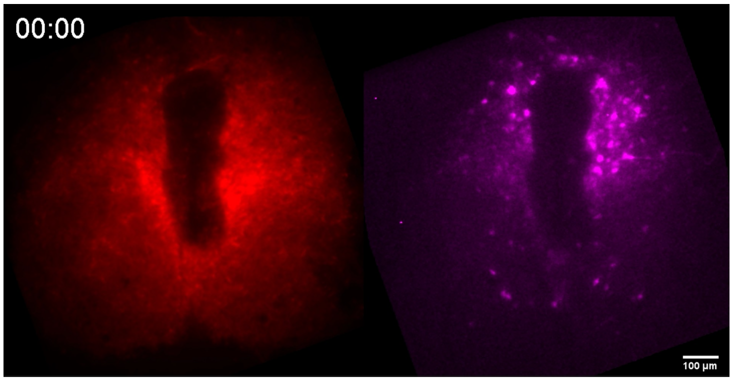


**Movie S1 (separate file). Movie S1. Circadian oscillations of Syn-jRCaMP1a and Rbm3-Luc monitored in SCN slices.** Left: neuronal calcium, right: Rbm3-Luc. Sample is imaged every 30 minutes for five consecutive days at 37°C.

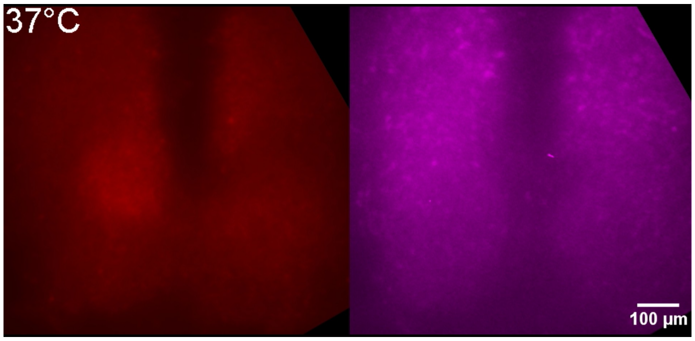


**Movies S2 (separate file). related to Figure 5 and S5. Reduced Bmal1 expression irreversibly impairs spatiotemporal organization of Rbm3-luc and SCN circuit activity with temperature changes.** Neuro-Bmal1$^{red}$ shows normal circadian spatiotemporal organization of neuronal calcium and Rbm3-Luc during four days of baseline recording at 37°C, which this is impaired when temperature is decreased to 32°C. Reverting temperature back to 37°C does not restore the spatiotemporal organization of neuronal calcium and Rbm3-Luc signals.

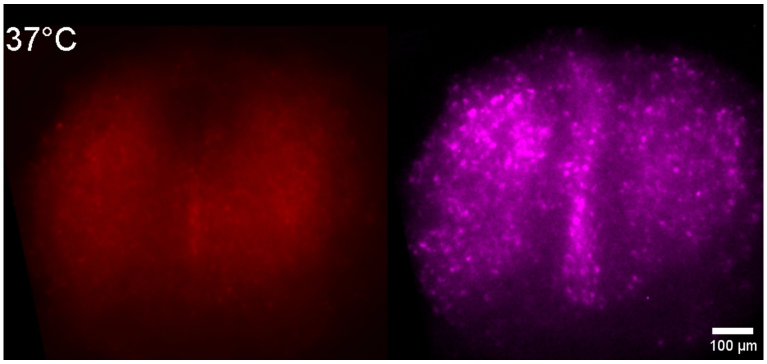


**Movies S3 (separate file). related to Figure 5 and S5. Change in temperature does not affect spatiotemporal organization in wild-type SCN slices.** In contrast to the neuro-Bmal1$^{red}$ sample, spatiotemporal organization of the SCN circuit is preserved with changes of temperature in WT SCN slices

## Supplementary References


1. Y. Liu, *et al.*, Cold-induced RNA-binding proteins regulate circadian gene expression by controlling alternative polyadenylation. *Sci Rep* **3**, 2054 (2013).

2. J. Pilotte, E. E. Dupont-Versteegden, P. W. Vanderklish, Widespread Regulation of miRNA Biogenesis at the Dicer Step by the Cold-Inducible RNA-Binding Protein, RBM3. *PLOS ONE* **6**, e28446 (2011).

3. F. Smart, *et al.*, Two isoforms of the cold-inducible mRNA-binding protein RBM3 localize to dendrites and promote translation. *J Neurochem* **101**, 1367–1379 (2007).

4. S. M. Sertel, M. S. von Elling-Tammen, S. O. Rizzoli, The mRNA-Binding Protein RBM3 Regulates Activity Patterns and Local Synaptic Translation in Cultured Hippocampal Neurons. *J. Neurosci.* **41**, 1157–1173 (2021).
